# Does pleural fluid appearance really matter? The relationship between fluid appearance and cytology, cell counts, and chemical laboratory measurements in pleural effusions of patients with cancer

**DOI:** 10.1186/1749-8090-5-63

**Published:** 2010-08-18

**Authors:** Bulent Ozcakar, Carlos H Martinez, Rodolfo C Morice, Georgie A Eapen, David Ost, Mona G Sarkiss, Hsienchang T Chiu, Carlos A Jimenez

**Affiliations:** 1 Department of Pulmonary Medicine, The University of Texas M. D. Anderson Cancer Center, 1400 Hermann Pressler Dr, Unit 1462, Houston, TX, 77030-4008, USA; 2 Departments of Internal Medicine and Preventive Medicine and Community Health, The University of Texas Medical Branch, Galveston, TX, USA; 3 Department of Anesthesia and Preoperative Medicine, The University of Texas M. D. Anderson Cancer Center, Houston, TX, USA; 4 Division of Pulmonary Medicine, The University of Texas Southwestern Medical Center, Dallas, TX, USA

## Abstract

**Background:**

Previous reports have suggested that the appearance of pleural effusions (i.e., the presence or absence of blood) might help to establish the etiology of the effusions. This study explores the relationship between pleural fluid appearance and the results of chemical and cytological analyses in a group of patients with recurrent symptomatic pleural effusions and a diagnosis of cancer.

**Methods:**

Medical records were reviewed from all 390 patients who were diagnosed with cancer, who underwent thoracentesis before placement of an intrapleural catheter (IPC) between April 2000 and January 2006. Adequate information for data analysis was available in 365 patients. The appearance of their pleural fluid was obtained from procedure notes dictated by the pulmonologists who had performed the thoracenteses. The patients were separated into 2 groups based on fluid appearance: non-bloody and bloody. Group differences in cytology interpretation were compared by using the chi square test. Cellular counts, chemical laboratory results, and survival after index procedure were compared by using the student's t test.

**Results:**

Pleural fluid cytology was positive on 82.5% of the non-bloody effusions and on 82.4% of the bloody ones. The number of red blood cells (220.5 × 10^3^/μL vs. 12.3 × 10^3^/μL) and LDH values (1914 IU/dl vs. 863 IU/dl) were statistically higher in bloody pleural effusions.

**Conclusion:**

The presence or absence of blood in pleural effusions cannot predict their etiology in patients with cancer and recurrent symptomatic pleural effusions.

## Background

Pleural effusions are a common problem in cancer patients. In a postmortem series published by Rodriguez-Panadero and colleagues[1], there was evidence of malignant pleural involvement in 28% of patients with one or more malignant tumors, of whom approximately one-half presented with a pleural effusion. The incidence of malignant pleural effusions in the United States approaches 150,000 cases annually[2].

For patients presenting with clinical signs of a pleural effusion, the primary diagnostic tools include roentgenographic studies of the chest and a thoracentesis. Initial information about the pleural effusion comes from the color and appearance of the fluid obtained during thoracentesis. Additional information concerning the inflammatory characteristics of the fluid is obtained later, using indicators such as white and red blood cell counts, and chemical laboratory values including glucose, protein, LDH, amylase, and cholesterol [3-5].

A small number of previous publications have attempted to find an association between pleural fluid appearance and cytological diagnosis in patients presenting with a pleural effusion. Two studies on patients without a prior diagnosis of cancer reported an association between bloody effusions and the presence of malignant cells on pleural fluid cytology [6,7]. In a study of patients with parapneumonic or infection-related effusions, a fluid that was free-flowing and non-purulent indicated an infection that could be treated with antibiotics alone, whereas a fluid with a purulent appearance indicted the need for drainage of the affected pleural cavity[8].

To our knowledge, there are no publications in the medical literature evaluating the relationship between fluid appearance and the results of cytological evaluation and chemical laboratory testing of pleural effusions in patients with cancer. We therefore conducted a retrospective study in a population of cancer patients with a high pretest probability of having a malignant pleural effusion (MPE) to answer two questions: Is there any relationship between pleural fluid appearance and the presence of malignant cells on pleural fluid cytology? Does fluid appearance correlate with cellular counts or chemical laboratory measurements in pleural effusions?

## Methods

### Data extraction

This was a cross-sectional study in a group of patients with symptomatic pleural effusion and a previous diagnosis of cancer, who had a thoracentesis prior to placement of an indwelling pleural catheter (IPC). The protocol and a waiver of informed consent were approved by the University of Texas M. D. Anderson Cancer Center Institutional Review Board.

Between April 2000 and January 2006, 390 patients received a thoracentesis as part of their evaluation prior to IPC insertion. Of this group, 365 patients had a complete description of the procedure in their medical records, including appearance of the fluid, as well as results of cytology, cell counts, and chemical laboratory analysis.

Medical records were reviewed to collect data on age, gender, type of primary malignancy, number of previous thoracentesis procedures, and results of the index procedure. The index procedure was defined as the thoracentesis performed immediately before insertion of the IPC. Pleural fluid chemical laboratory reports were reviewed to retrieve levels of glucose, total protein, LDH, cholesterol, and triglycerides. Cytology results, white blood cell count with differential, red blood cell count, and survival after the index procedure were also recorded. Information on pleural fluid appearance was extracted from procedure notes available in the electronic medical record, dictated by the pulmonary faculty who had performed the thoracentesis. We separated patients into two groups based on fluid appearance: bloody and non-bloody. No purulent effusions were observed in our group.

### Analysis

Age, cell counts, and the results of chemical laboratory analysis of pleural fluids are presented as mean values with standard deviations and 95% confidence intervals (95% CI) of the mean. Gender, type of primary malignancy, fluid appearance, and cytology results are presented as frequencies and proportions. Differences in pleural fluid cell counts, chemical laboratory parameters, survival after index procedure, and cytology interpretation were compared between fluid appearance groups. Group differences for cytology interpretation were compared using the chi square test. Group differences in cellular counts, chemical laboratory results, and survival after index procedure were compared using student's t test and 95% CI of the mean.

## Results

Demographics, type of primary malignancy, and pleural fluid appearance are shown in Table 1. Almost half of the patients had lung cancer as a primary malignancy, followed by breast cancer (21%) and gastrointestinal cancer (6%). Effusions were described as non-bloody in 206 patients (56.4%) and bloody in 159 patients (43.6%). Cytology was positive in 82.5% of the patients. Red blood cell count and LDH were the only values significantly higher in bloody effusions (Table 2). There was no significant association between cytology results and pleural fluid appearance at any primary tumor type (Table 3).

**Table 1:** Demographic and clinical characteristics

Gender, n (%)	
Male	165 (45.2%)
Female	200 (54.8%)
**Age**	
Years, mean (SD)	58.7 (12.7)

**Tumor type, n (%)**	
Lung	177 (48.5%)
Breast	76 (20.8%)
Gastrointestinal	23 (6.3%)
Leukemia	17 (4.7%)
Lymphoma	16 (4.4%)
Other tumors	56 (15.3%)

**Fluid appearance, n (%)**	
Non-bloody	206 (56.4%)
Bloody	159 (43.6%)

**Cytology, n (%)**	
Positive	301 (82.5%)
Negative	64 (17.5%)

**Table 2:** Chemical measurements, cellular characteristics, and survival as a function of pleural fluid appearance.

Characteristics	Pleural Fluid Color		
	
	Non-bloody n = 206	Bloody n = 159	p value
**Primary malignancy**, **No. (%)**			
Lung	107 (51.9%)	70 (44.0%)	
Breast	45 (21.8%)	31 (19.5%)	
Gastrointestinal	11 (5.4%)	12 (7.5%)	
Leukemia	6 (2.9%)	11 (6.9%)	
Lymphoma	10 (4.9%)	6 (3.8%)	
Other tumors	27 (13.1%)	29 (18.3%)	

**Previous thoracentesis**			
Mean (95% CI)	1.7 (1.6 - 1.9)	1.8 (1.7 - 1.9)	NS

**Mean survival**			
Days (95%CI)	176.9 (141.2 - 212.6)	130.8 (101.0 - 160.6)	NS

**Fluid chemistry, Mean (95%CI)**			
Glucose (mg/dl)	102.9 (96.6 - 109.2)	93.5 (86.5 - 100.4)	<0.05
Protein (g/dl)	3.8 (3.7 - 4.0)	4.1 (3.9 - 4.3)	NS
LDH (IU/dl)	863.4 (680.8 - 1046.1)	1913.6 (1454.1 - 2373.1)	<0.01
Cholesterol (mg/dl)	70.8 (66.3 - 75.3)	73.1 (67.8 - 78.4)	NS

**Cellular characteristics**, **Mean (95%CI)**			
Mean WBC 10^3^/μL	0.7 (0.6 - 0.9)	1.6 (0.9 - 2.3)	<0.01
Mean RBC 10^3^/μL	12.3 (7.1 - 17.5)	220.5 (81.8 - 359.2)	<0.01
PMN cells (%)	15.0 (12.0 - 18.0)	20.5 (16.5 - 24.5)	<0.05
Mononuclear cells (%)	64.0 (60.0 - 68.0)	60.8 (56.3 - 65.3)	NS

**Cytology**, **No. (%)**			
Positive	170 (82.5%)	131 (82.4%)	NS
Negative	36 (17.5%)	28 (17.6%)	NS

**Table 3:** Cytology results as a function of pleural fluid appearance and tumor type.*

		Pleural Fluid Appearance	
		
Characteristics		Non- Bloody n = 206	Bloody n = 159
**Primary malignancy, No. (%)**			

Lung	+ Cytology	93 (86.9%)	64 (91.4%)
	
	- Cytology	14 (13.1%)	6 (8.6%)

Breast	+ Cytology	40 (88.9%)	28 (90.3%)
	
	- Cytology	5 (11.1%)	3 (9.7%)

Gastrointestinal	+ Cytology	11 (100.0%)	11 (91.7%)
	
	- Cytology	0 (0.0%)	1 (8.3%)

Leukemia	+ Cytology	3 (50.0%)	8 (72.7%)
	
	- Cytology	3 (50.0%)	3 (27.3%)

Lymphoma	+ Cytology	4 (40.0%)	6 (100.0%)
	
	- Cytology	6 (60.0%)	0 (0%)

Other tumors	+ Cytology	19 (70.4%)	14 (48.3%)
	
	- Cytology	8 (29.6%)	15 (51.7%)

*Statistical comparisons were not significant.

## Discussion

Pleural fluid cytology was positive on 82.5% of the non-bloody effusions and on 82.4% of the bloody ones. The number of red blood cells (220.5 × 10^3^/μL vs. 12.3 × 10^3^/μL) and LDH values (1914 IU/dl vs. 863 IU/dl) were statistically higher in bloody pleural effusions. The presence or absence of blood in pleural effusions cannot predict their etiology in patients with cancer and recurrent symptomatic pleural effusions.

Our study was subject to the limitations of any retrospective study. However, the potential effect of any information bias is minimal, as we used pathologic and laboratory data that were not influenced by the clinician or the personnel conducting the research. Of more concern is the external validity of our data. Our results are applicable only to patients with known primary extrapleural malignancies presenting with a recurrent symptomatic pleural effusion, not to the general patient population with a lower pretest probability of having a MPE. In addition, our patient sample did not include any findings of "purulent" fluids, so our conclusions do not encompass this possibility.

It would be extremely useful if an easily assessed parameter like pleural fluid appearance could be prospectively used to identify patients with positive cytology or to estimate the inflammatory or tumor burden on the pleural space in patients with a previous diagnosis of cancer. It would potentially reduce the number of interventions performed in patients with MPE prior to definitive therapy. Timely identification of the inflammatory pleural response would also be of great interest, as some authors have hypothesized that the results of chemical pleurodesis could be predicted using cellular and chemical characteristics of the pleural fluid[9,10].

Several published studies using patients presenting with pleural effusions without a prior history of cancer have found a correlation between bloody pleural effusion and malignancy. In a series of 163 patients with large or massive pleural effusions, Porcel and Vives [11] reported significantly higher RBC counts in patients with MPEs compared with patients with nonmalignant effusions (median value 18 × 10^9 ^cells/L versus 2.7 and 10^9 ^cells/L, respectively; p < 0.001).

In another prospective study of 334 consecutive patients with chronic pleural effusion reported by Martensson and colleagues, 86% of the 44 bloody fluids and 57% of the 65 blood-tinged fluids were cancerous on cytology or biopsy (p < 0.01) [6]. Villena and coworkers found that the presence of a bloody fluid slightly increased the probability of a malignant pleural effusion (Odds Ratio = 1.73). In her series of 715 patients, 47% of the bloody effusions were MPE. The authors concluded that fluid appearance should not be overemphasized as a diagnostic tool [7]. However, our study does not support an extrapolation of these reports to patients with a known history of cancer. In this study, we found no association between pleural fluid appearance and chemical laboratory analysis, cell counts (except for LDH and RBC), or presence of malignant cells on cytology in patients with a previous diagnosis of cancer and a high pretest probability of having MPE.

In our results, although 82.5% of bloody fluids showed a positive cytology, this percentage was not significantly different than that observed in the non-bloody fluids (82.4%). However, our population comprised patients with a prior diagnosis of cancer and a higher pretest probability of having an MPE. So, while bloody pleural effusions may be suggestive of positive cytology in a general population of patients presenting with pleural effusions, it does not appear to be useful in this regard in cancer patients.

## Conclusions

In summary, we found no relation between pleural fluid appearance, chemical laboratory parameters, cytological results, or survival in patients with cancer presenting with recurrent symptomatic pleural effusion. Therefore, pleural fluid appearance should not be used as an indicator of MPE in this patient group.

Further studies that include measurements of more specific inflammatory biomarkers are required to determine if pleural fluid appearance can predict the degree of intrapleural inflammatory response as it could be one of the factors related with pleurodesis success [12,13].

## Competing interests

The authors declare that they have no competing interests.

## Authors' contributions

BO: Conception and design of the study, collection and assembly of data, data analysis and interpretation, manuscript writing and final approval of manuscript. CHM: Conception and design of the study, collection and assembly of data, data analysis and interpretation, manuscript writing and final approval of manuscript. RCM: Conception and design of the study, manuscript writing and final approval of manuscript. GAE: Conception and design of the study, manuscript writing and final approval of manuscript. DO: Conception and design of the study, manuscript writing and final approval of manuscript. MS: Conception and design of the study, manuscript writing and final approval of manuscript. HTC: Conception and design of the study, manuscript writing and final approval of manuscript. CAJ: Conception and design of the study, provision of study materials and patients, data analysis and interpretation, manuscript writing and final approval of manuscript.
